# Poly(ε-Caprolactone)/Poly(Glycerol Sebacate) Composite Nanofibers Incorporating Hydroxyapatite Nanoparticles and Simvastatin for Bone Tissue Regeneration and Drug Delivery Applications

**DOI:** 10.3390/polym12112667

**Published:** 2020-11-12

**Authors:** Abdelrahman I. Rezk, Kyung-Suk Kim, Cheol Sang Kim

**Affiliations:** 1 Department of Bionanosystem Engineering, Graduate School, Jeonbuk National University, Jeonju, Jeonbuk 561-756, Korea; rezk@jbnu.ac.kr; 2 Department of Bionanotechnology and Bioconvergence Engineering, Graduate School, Jeonbuk National University, Jeonju, Jeonbuk 561-756, Korea; 3 Department of Molecular Biology, College of Natural Sciences, Jeonbuk National University, Jeonju 561-756, Korea; 4 Division of Mechanical Design Engineering, Jeonbuk National University, Jeonju, Jeonbuk 561-756, Korea

**Keywords:** electrospinning, biomaterial, composite nanofiber, drug delivery, bone tissue regeneration

## Abstract

Herein, we report a drug eluting scaffold composed of a composite nanofibers of poly(ε-caprolactone) (PCL) and poly(glycerol sebacate) (PGS) loaded with Hydroxyapatite nanoparticles (HANPs) and simvastatin (SIM) mimicking the bone extracellular matrix (ECM) to improve bone cell proliferation and regeneration process. Indeed, the addition of PGS results in a slight increase in the average fiber diameter compared to PCL. However, the presence of HANPs in the composite nanofibers induced a greater fiber diameter distribution, without significantly changing the average fiber diameter. The in vitro drug release result revealed that the sustained release of SIM from the composite nanofiber obeying the Korsemeyer–Peppas and Kpocha models revealing a non-Fickian diffusion mechanism and the release mechanism follows diffusion rather than polymer erosion. Biomineralization assessment of the nanofibers was carried out in simulated body fluid (SBF). SEM and EDS analysis confirmed nucleation of the hydroxyapatite layer on the surface of the composite nanofibers mimicking the natural apatite layer. Moreover, in vitro studies revealed that the PCL-PGS-HA displayed better cell proliferation and adhesion compared to the control sample, hence improving the regeneration process. This suggests that the fabricated PCL-PGS-HA could be a promising future scaffold for control drug delivery and bone tissue regeneration application.

## 1. Introduction

During recent decades, tissue engineering (TE) strategies have been considered promising in the field of medicine, aiming to develop biological substitutes that restore tissue functions [1,2]. Medical advances have opened a new era to welcome the increase in world population demographics. However, increased aging people pose new trials and emphasize the necessity for utilization of new approaches including nanotechnology, composite biomaterials and stem cell therapy to enhance and repair bone tissue lost [3,4]. To meet this demand, drug eluting scaffolds serve as templates for the establishment of the vascular system and induce cell growth in tissue engineering [5,6]. Recent studies have suggested the potential to use composite and drug eluting implants (naturally or synthetic derived matrices) for bone regeneration [1,7,8,9]. Pleiotropic bone substitutes with both osteogenic and angiogenic potential are gaining excessive attention for treatment of bone diseases or loss caused by trauma, tumors, infections or genetic malformations [7,10,11].

Poly(glycerol sebacate) (PGS) is a novel polyester, biodegradable elastomer polymer owing to the properties of its starting materials that are similar to collagen, low-cost and prepared by a simple polycondensation of glycerol and sebacic acid to generate a viscous prepolymer [12,13]. PGS has linear degradation kinetics that can be controlled and make it suitable for drug delivery. Moreover, these bio-products are harmless and well tolerated in the body; consequently, the US Food and Drug Administration has permitted them for therapeutic applications [14,15]. Moreover, PGS displays properties similar to collagen and elastin that make it suitable for tissue regeneration applications. PGS itself is not able to be electrospun; therefore, many trials has been conducted to form PGS into nanofibers to form scaffold-mimicking ECM such as other polymers blended to provide mechanical support [16,17]. Electrospinning seems to be a proper method to process PGS. However, after curing, its crosslinks cannot be dissolved in organic solvents easily. Therefore, PGS itself is not able to be electrospun. poly(ε-caprolactone) (PCL) is a semicrystalline hydrophobic polymer that is widely used in electrospinning processes. However, it shows poor cell compatibility due to its hydrophobicity. Other studies in the literature have modified PCL with natural polymers such as collagen to improve its cell attachment, spreading and proliferation. The addition of PCL increased the viscosity of the polymer blend, making PGS suitable for electrospinning [16,18]. Taken together, this study shows that the blending of PGS and PCL was a useful strategy to complement their individual properties, such as mechanical properties and biological behavior, to increase the scaffold performance for tissue regeneration applications [19]. Various studies have reported on the fabrication of different inorganic nanoparticles such as bioactive glass [20,21], tricalcium phosphate [22] and hydroxyapatite [23] and their incorporation in natural or synthetic polymers such as the collagen, chitosan, polycaprolactone, poly lactic acid and polyhydroxyalkanoates. Among these inorganics, synthetic Hydroxyapatite nanoparticles (HANPs) are the most widely used bioceramic material with similar composition and morphology to the inorganic component of natural bone [11,24]. HANPs are highly biocompatible and biodegradable, showing excellent identity with the inorganic part of vertebrate bone. Furthermore, it has been used for various biomedical applications, including scaffolds for controlled drug delivery and tissue engineering. However, due to its brittleness and low strength, HANPs are “limited to the reconstruction of non-stress-bearing bone” [25,26].

It would be highly desirable to combine electrospun nanofibers with HANPs to gain the benefits of both materials: the injectability, bioactivity and mechanical properties from HANPs, and the high surface area for cell attachment and fiber reinforcement from the electrospun fibers. Several approaches have focused on local delivery strategies of simvastatin (SIM) to allow sufficient dosage at the site of action and limit systemic side effects of oral usage including liver toxicity and myositis [27,28]. Locally delivered SIM is reported to have a positive effect on bone tissue regeneration and the treatment of periodontal disease as well as enhance osseointegration around bone implants [11,29]. However, high doses of statins may lead to an increase in statin-related adverse effects. For example, a previous study revealed that a dose of 2.2 mg of simvastatin in methylcellulose gel loaded into a polylactide significantly improved bone healing, but the high dose resulted in ulceration and edema [30].

In this work, we fabricated elastomeric nanofibrous scaffolds using blends of non-acrylated PGS prepolymer with biodegradable PCL by taking advantage of local drug delivery potential of nanofibers with SIM as an alternative to expensive growth factors, and demonstrating the ability of drug eluting scaffold to support bone tissue regeneration. The present study aims to fabricate a novel electrospun PCL/PGS composite fibrous structure incorporating HANPs and SIM. Moreover, the fiber mats were investigated regarding their morphology, drug release, biomineralization and cytocompatibility. The results confirmed that adding HANPs provides a suitable environment for late osteogenic differentiation and bio mineralization, while SIM supports the initial attachment and proliferation of pre-osteoblasts cells.

## 2. Experimental

### 2.1. Materials and Methods

Poly (ε-caprolactone) (PCL, *M*_w_ = 70,000–90,000), ammonium phosphate dibasic ((NH_4_)_2_HPO_4_), sebacic acid, Hank’s balanced solution, glycerol and simvastatin were purchased from Sigma Aldrich (Seoul, Korea). Calcium nitrate tetrahydrate (Ca(NO_3_)_2_·4H_2_O, Alfa Aesar Company, Seoul, Korea), aqueous ammonia solution, N, N Dimethylformamide (DMF, 99.5% Samchun, Seoul, Korea) and tetrahydrofuran (THF, 99.8% Samchun) were used as received.

**Scheme 1:**
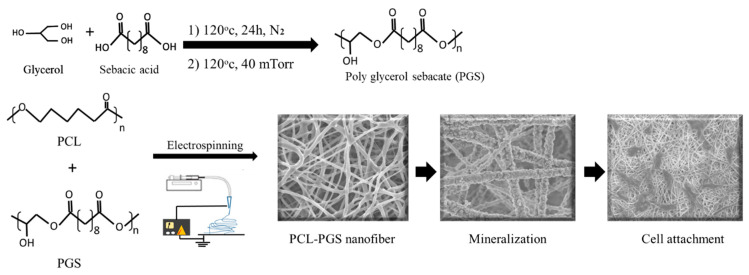
The condensation reaction of poly glycerol sebacate, and electrospinning process to make PCL-PGS nanofiber.

The PGS prepolymer was synthesized according to previously published methods [31] with minor modification. Briefly, a mixture of glycerol and sebacic acid (1:1) was reacted at 120  °C under nitrogen for 24 h. Then, the pressure was reduced to 40 mTorr and the reaction was held at 120  °C for another 48 h to obtain the viscous PGS prepolymer as shown in Scheme 1.

Hydroxyapatite nanoparticles (HANPs) were synthesized using a wet chemical precipitation method, as reported previously by our group [23].

### 2.2. Solution Preparation for Electrospinning

The composite nanofiber mats were fabricated using the custom made electrospinning setup. In this study, the PCL-PGS composite nanofibers were prepared by electrospinning, 12 wt.% PCL was dissolved in DMF-THF (1:1), and mixed with PGS by ratio of (1:1) and stirred at room temperature overnight. Then 5 wt.% of HANPs of the total polymer weight were added to the solution and ultra-sonicated for 15 min at 60 °C before electrospinning to make PCL-PGS-HA composite nanofibers. For drug loaded samples, SIM was added to the composite solution immediately before electrospinning, sonicated for 10 min (SIM concentration was 5 wt.% of the polymer weight) and given the name PCL-PGS-HA-SIM. Prior to electrospinning, we optimized the weight percentage of PCL (12 wt.%), HA (5 wt.%) and SIM (5 wt.%) based on spinnable solution to fabricate relatively uniform diameter and bead-free electrospun nanofibers with porous networks. The electrospinning process was carried out at flow rate of 1 mL/h using high DC voltage of 17 KV using needle gauge 21. The distance between the rotating drum and nozzle was 15 cm, and the electrospun nanofibers were collected on a polyethylene sheet, then dried in a vacuum oven.

### 2.3. Characterizations

The morphology of the electrospun composite nanofiber were analyzed by field-emission scanning electron microscopy (FE-SEM, Carl Zeiss supra-40 VP, Jena, Germany). Fiber diameters were measured using the software Image J (NIH, Bethesda, MD, USA). The bonding configurations of the composite mats were characterized by Spectrum GX Fourier transform infrared (FT-IR) spectroscopy (Perkin Elmer, Waltham, MA, USA). FT-IR spectra were measured in the range (400–4000) cm^−1^.

The thermal stability of scaffolds was studied using a Q50 TGA device (TA Instruments, New Castle, DE, USA). The samples with 10 mg were heated from room temperature to 800 °C at a rate of 10 °C/min under a nitrogen atmosphere to avoid oxidation of the nanofibers mats. All other experiments are described in the electronic Supplementary Materials.

## 3. Results

### 3.1. Morphological Characterization and Physiochemical Properties

An electrospinning technique was used to produce a drug eluting nanofiber that mimicked the structure of natural ECM in terms of architecture, which is among the methods for suboptimal outcomes in biomedical applications.

**Figure 1:**
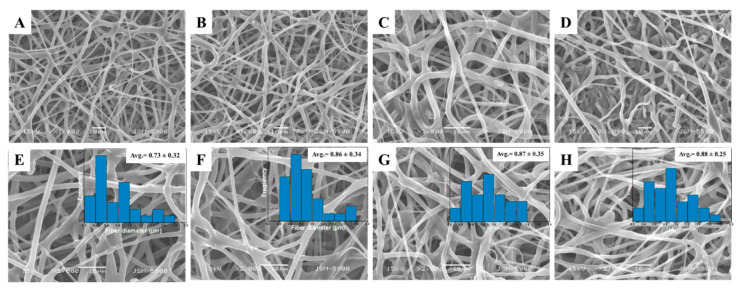
SEM images of (**A**,**E**) PCL, (**B**,**F**) PCL-PGS, (**C**,**G**) PCL-PGS-HA, (**D**,**H**) PCL-PGS-HA-SIM at two different magnification point. (Scale bar = 10 µm). Inset plots represent fiber diameter distribution histograms and their average value of fiber diameter.

PCL-PGS-HA-SIM was successfully fabricated and the morphology of the nanofibers was observed through SEM. Figure 1A–H show selected SEM images that display the morphology of the nanofibrous mat of (A) PCL, (B) PCL-PGS, (C) PCL-PGS-HA and (D) PCL-PGS-HA-SIM. The selected images show bead-free fibrous morphology with uniform diameter that mimics the natural extracellular matrix (ECM). As mentioned previously, PCL is a semi-crystalline, biodegradable polymer which has been extensively used in bone tissue regeneration applications. Based on the previous literature, the utilization of PGS is beneficial to enhance the properties of PCL as composite nanofibers scaffolds, as well as a carrier for nanoparticles and drug release. The fiber diameter significantly increased with the addition of PGS to PCL (0.73 ± 0.32 μm), to reach values of 0.86 ± 0.34 μm and 0.87 ± 0.35 μm, 0.88 ± 0.25 μm for PCL-PGS-HA and PCL-PGS-HA-SIM, respectively. Previous literature indicated that adding PGS to PCL increased the nanofiber diameter due to the high concentration of total polymer solution. The addition of HANPs did not significantly change the average fiber diameter. Moreover, the distribution of fiber diameter inset plots reported in Figure 1 shows that their maximum and minimum values fluctuate more noticeably in samples containing HANPs. This effect is likely caused by the increased suspension conductivity in the presence of HANPs.

The chemical characterization of the composite PCL-PGS nanofibers in comparison with PCL nanofibers as well as with the PGS pre-polymer was performed by XRD and FTIR analysis (Figure S1). XRD spectra of PCL nanofibers show the presence of two peaks at 21.5° and 23.7°, while the PGS polymer exhibits three peaks at 19.4°, 21.4° and 23.1°. The XRD spectra for the composite PCL/PGS nanofibers present three peaks including the two major peaks of the PCL spectra and a peak at 19.4° that can be assigned to the PGS portion, thereby confirming the presence of both PCL and PGS polymers in their constitution.

FTIR spectroscopy was used to confirm the chemical interaction between PCL and PGS in the electrospun scaffolds. Both PCL and PGS nanofibers showed very similar major IR peaks at approximately 2927 cm^−1^ CH_2_ asymmetric stretching, 2855 cm^−1^ CH_2_ symmetric stretching, 1722 cm^−1^ carbonyl bond stretching, 1127 cm^−1^ of C–O and C–C stretching in the amorphous phase and 1166 cm^−1^ (carbon–oxygen bond stretching) and at 1293 cm^−1^ in the crystalline part. These stretching modes display a stronger intensity band corresponding to the amorphous phase, and consistent with the XRD analysis. Despite being slightly masked by the higher amplitude of other peaks, the PGS spectra showed an additional hydroxyl group peak at 3445 cm^−1^, which was not present in PCL. Changes are observed for the hydroxyl signal between 3300 and 2500 cm^−1^, which indicates an increase in hydroxyl groups in the blend samples in comparison to pure PCL. The PCL-PGS nanofibers presented all the mentioned peaks, confirming the integration of PGS into the composite nanofibers.

Thermal behavior determines the physical properties of the tissue engineering scaffold, such as mechanical stability and degradation. Therefore, thermal behavior is an important characteristic property to be extensively studied while fabricating any new polymer composite. To gain deeper insight into the thermal properties of the electrospun PCL-PGS scaffolds, we performed TGA and DSC tests on the samples. As shown in Figure S1, Pure PCL fibers exhibited a single-stage weight loss at 374 °C and pure PGS material started to decompose at approximately 410 °C. However, PCL-PGS Figure S1 showed a weight loss that began around 367 °C that was lower than that of pure PCL. As expected, the PCL-PGS nanofibers exhibit two distinct (an endothermic peak at about 60.14 °C related to the melting point) melting temperatures (Tm) (Tm–PCL = 60.14 °C and Tm–PGS = 43 °C). The endothermic peak for PGS-PCL was slightly left-shifted, and the post-treated fiber exhibited endothermic peaks at around 60.89 °C. Overall, the results indicated that PCL and PGS were partly miscible after the electrospinning process.

### 3.2. Drug Release

**Figure 2:**
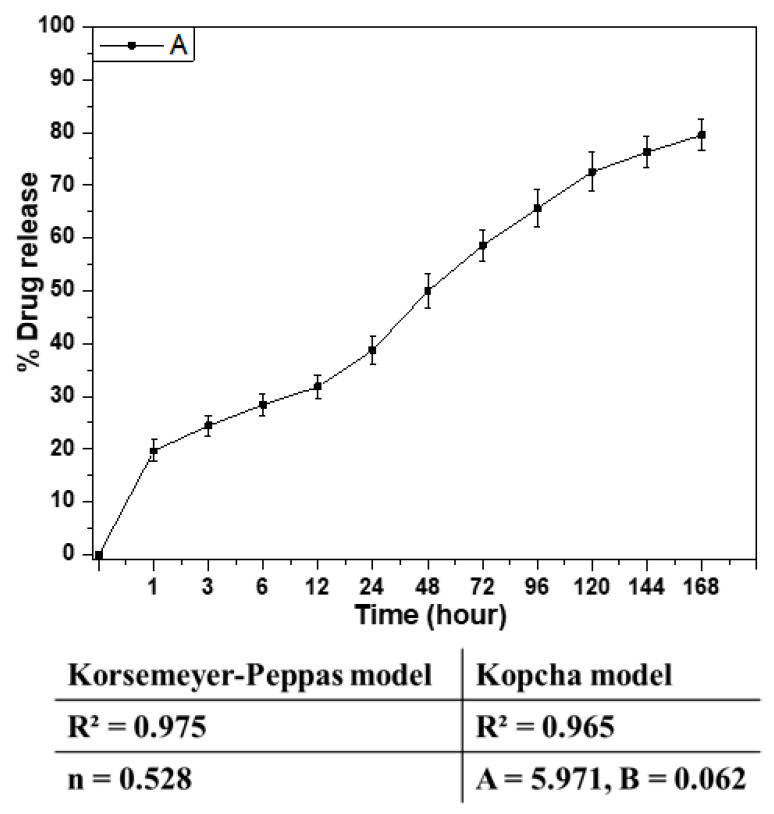
Cumulative in vitro drug release of (A) PCL-PGS-HA-SIM composite nanofiber in PBS. The table shows the in vitro release kinetics of SIM in PBS.

SIM was loaded into the PCL-PGS-HA solution to produce PCL-PGS-HA-SIM nanofibers. PCL-PGS-HA-SIM nanofibers mat was assessed for the in vitro release studies of SIM during 7 days as shown in Figure 2, the SIM release behavior was characterized by an initial burst release around 20% until 24h, followed by a gradually slow and nearly linear release. As shown in Figure 2, during the 7 days of the in vitro study, a total of 79.5% SIM was released from the PCL-PGS-HA-SIM. The drug release from nanofibers mat can be described as after the nanofiber soaked in water, the fibers will absorb some water from the dissolution media and then change the dimension and physicochemical properties of the nanofibers, which will result in facilitating the drug release from nanofiber matrices. Therefore, we revealed that the initial 20% release of SIM from a nanofibrous mat could be attributed to the amount of the drug near the nanofibrous surface. Moreover, the ability of drug molecules to accumulate on the surface of the nanofibers during the electrospinning was explained in previous literature [29,32] and attributed to the low molecular weight and affinity of drug particles to the aqueous solvent.

The Korsemeyer–Peppas model is essential to understand the release behavior and kinetics from polymeric materials. The drug release data are fitted to the Korsemeyer–Peppas model and Kopcha model. On the basis of the exponent release value (n), this model describes that either the release behavior follows Fickian diffusion or not. In this study, n = 0.528 (0.5 < n < 1), indicating a non-Fickian (anomalous) diffusion mechanism consisting of a combination of diffusion and erosion mechanisms. The in vitro release data from PCL-PGS-HA-SIM were summarized in terms of mathematical model correlation coefficient, reported in Figure 2.

The Kopcha model is also often used to quantify the relative effects of diffusion and polymer relaxation on drug release. The calculated value of ”A” and ”B” specifies both mechanism (diffusion and erosion). The diffusional exponent A = 5.971, and the erosional constant B = 0.062. It is clear that the value of ”A” is greater than ”B”, which indicates that in this medium, drug release is quantitatively controlled by a diffusion process rather than an erosion process.

### 3.3. In Vitro Biomineralization Study

**Figure 3:**
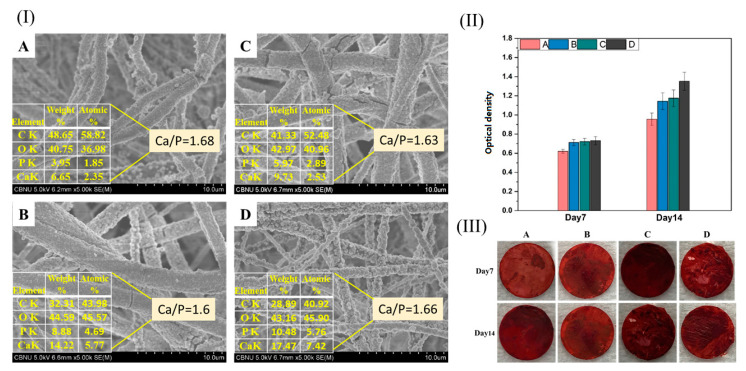
(**I**) SEM images and inset tables of EDS analysis after incubation in SBF solution for 10 days. (**II**) Absorbance shown by Alizarin Red S extracted from the stained calcium deposits on the surface of different nanofibrous mats treated with SBF solution. (**III**) The inset shows selected digital images of different nanofibrous mats. (**A**) PCL, (**B**) PCL-PGS, (**C**) PCL-PGS-HA, (**D**) PCL-PGS-HA-SIM.

Figure 3 shows the deposition and nucleation of hydroxyapatite on the surface of the electrospun nanofibrous mats as a component. During the incubation periods in the SBF solution, hydroxyapatite is formed and deposited as Ca/P minerals on the surface of different samples, as shown in the FESEM images.

The SEM images show that the Ca and P minerals cluster on the surface of PCL nanofibrous scaffolds, while for composite nanofiber massive ions, covered its surface. The clusters of Ca and P minerals are bulky with relatively uniform size, indicating appropriate nucleation of calcium and phosphate ions on the PCL-PGS-HA-SIM composite nanofibrous.

EDS analysis of different nanofibrous mats confirmed the presence of Ca and P ions on the surface of the nanofibers demonstrating the ability of the fabricated scaffold to make an apatite-like layer. Based on EDS results, the ratio of Ca/P deposition on the surface of PCL, PCL-PGS, PCL-PGS-HA and PCL-PGS-HA-SIM nanofibrous mats was found to be 1.68, 1.63, 1.6 and 1.68, respectively. The obtained result revealed the ability of PCL-PGS-HA-SIM composite nanofibrous mat to make an apatite-like layer close to the natural layer.

Alizarin red staining was conducted to verify the biomineralization on different nanofibrous mats as osteogenic markers at the late stage, Calcium deposits on the 2D scaffold are an indication of successful ECM mineralization, an innate character of bone-like structures, and can specifically be stained bright orange-red using Alizarin Red S.

The OD of ARS extracted from stained cultures is shown in Figure 3. Furthermore, Figure 3 (III) shows that the color intensity for all samples after 14 days increased considerably compared with that after 7 days. Additionally, the relative absorbance value of the matrix mineralization cultured with PCL-PGS-HA and PCL-PGS-HA-SIM at 7 and 14 days was significantly higher compared to PCL due to the matrix mineralization of the composite nanofibers mat. The results indicated that the addition of HANPs and SIM could endorse extracellular matrix mineralization and hence the osteogenic differentiation. According to the ARS quantitative results in Figure 3 (II, III), PCL-PGS induces more calcium nodules than bare PCL PCL-PGS-HA, while PCL-PGS-HA-SIM has the highest Ca ion deposition and mineralization. Additionally, more significant regions of bright red color are observed on composite samples compared to the PCL sample, especially for PCL-PGS-HA and PCL-PGS-HA-SIM, from ARS staining for calcium-related clusters, indicating that the newly deposited ECM is calcified by SBF solution.

### 3.4. Cell Culture

**Figure 4:**
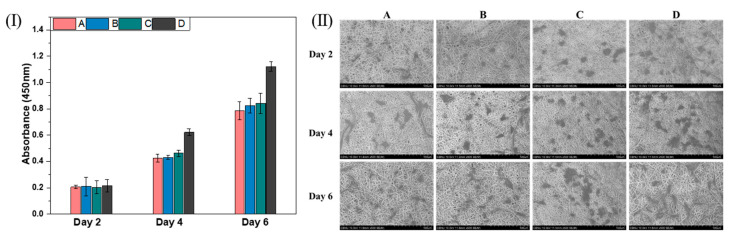
(**I**) CCK-8 assay show proliferation of MC3T3-E1 cells, (**II**) FE-SEM images of MC3T3-E1 cells cultured on different nanofibrous mats. The cells were seeded for 2, 4 and 6 days. (**A**) PCL, (**B**) PCL-PGS, (**C**) PCL-PGS-HA, (**D**) PCL-PGS-HA-SIM.

MC3T3E1 osteoblast cells were seeded on different composite nanofibrous mats to evaluate cell adhesion and proliferation on day 2, 4 and 6. Our results revealed that cells spread well on the surface of PCL-PGS-HA-SIM nanofibrous mat compared to the PCL sample as shown in Figure 4 (I and II). Different nanofiber samples displayed similar results of cell adhesion and spreading after day 2, while after day 6, the morphology of MC3T3-E1 cells seeded on the PCL-PGS-HA-SIM composite nanofibers indicated the existence of more associated layers of fully extended cells interconnected by the secretion of ECM. The deposition of hydroxyapatite on the electrospun nanofibers in a way that is similar to natural ECM resulted in successful mineralization and cell adhesion.

Furthermore, the cell viability of MC3T3-E1 on all samples was compared using the cell counting kit-8 (CCK-8) assay. Our results reveal that the differences in the cell viability on different samples confirm the effect of the composite nanofibers incorporating HANPs and SIM in the cellular response. Generally, cell viability in PCL-PGS and PCL-PGS-HA did not significantly increase during the culture time in comparison with PCL. However, the presence of SIM in the PCL-PGS-HA-SIM drug eluting sample increased the cell proliferation rate compared to PCL cultured for 6 days. This growth trend could be observed in the CCK-8 assay results shown in Figure 4. The optical density (OD) value and number of cells were the highest for the drug eluting sample revealing the osteogenic effect of SIM on cell growth.

## 4. Conclusions

Precisely bead-free PCL/PGS nanofibers incorporated with HANPs and SIM were successfully prepared in this study using the electrospinning technique. The as prepared composite nanofiber loaded HANPs, SIM shows excellent biomineralization inducing mineral deposition making a bone-like apatite layer on its surface. Korsemeyer–Peppas and Kpocha models were used to study drug release mechanism revealing drug release control by a diffusion rather than an erosion process. The in-vitro cell culture test revealed that the incorporation of HANPs, SIM into the composite nanofiber enhanced osteoblast cell growth, proliferation and adhesion, offering great potential for bone cell regeneration. The results of this study revealed that our composite nanofiber will be a promising future scaffold for bone tissue regeneration and drug delivery application by controlling SIM release, providing excellent biomineralization and improved bone cell proliferation.

## Supplementary Materials

The following are available online at https://www.mdpi.com/2073-4360/12/11/2667/s1, Figure S1: XRD patterns, FTIR spectra and TGA-DSC thermographs of different samples.
