# Supplementary material for: Poly(ε-Caprolactone)/Poly(Glycerol Sebacate) Composite Nanofibers Incorporating Hydroxyapatite Nanoparticles and Simvastatin for Bone Tissue Regeneration and Drug Delivery Applications
Source: Polymers (Basel). 2020 Nov 12;12(11):2667. doi: 10.3390/polym12112667 (PMC7697945; doi:10.3390/polym12112667)
Supplement: Supplementary file 1 [file polymers-12-02667-s001.pdf]

PCL/PGS Composite Nanofibers Incorporating Hydroxyapatite Nanoparticles and Simvastatin for Bone Tissue Regeneration and Drug Delivery Applications

Abdelrahman I. Rezk[1,2], Kyung-Suk Kim[3*], Cheol Sang Kim[2,4*]

[1] Department of Bionanosystem Engineering, Graduate School, Jeonbuk National University, Jeonju, Jeonbuk 561-756, Republic of Korea.

[2] Department of Bionanotechnology and Bioconvergence Engineering, Graduate School, Jeonbuk National University, Republic of Korea

[3] Department of Molecular Biology, College of Natural Sciences, Jeonbuk National University, Jeonju 561-756, Republic of Korea.

[4] Division of Mechanical Design Engineering, Jeonbuk National University, Jeonju, Jeonbuk 561-756, Republic of Korea.

***Corresponding authors:** chskim@jbnu.ac.kr (C.S.K.), sukkim@jbnu.ac.kr (K.S.K); Tel: +82 63 270 4284, Fax: +82 63 270 2460

2 Experimental

2.2 *In vitro* drug release study

Drug release from the electrospun nanofiber mat was determined by placing a known weight of nanofiber membrane with dimensions of (2×2 cm) into 10 mL of PBS (pH 7.4) and transferred into shaking incubator (SI-300R, Lab companion), (previously set at 37°C and 100 rpm). The amount of drug released was calculated at a wavelength 238 nm of maximum absorbance of simvastatin in PBS using a UV spectrophotometer, at predetermined time intervals, 3mL of PBS release media was used. The amount of simvastatin released was calculated by using a calibration curve constructed from the known simvastatin concentration and the calibration curve fits the Lambert and Beer's law:

$$\mathbf{y = ac + b}$$

Where y is absorbance and c designates the drug concentration.

Then the dissolve simvastatin at various time intervals were plotted as percentage release versus time.

Entrapment efficiency(%) = $\frac{\textit{weight of drug entrapped}}{\textit{amount of drug used}}$

The simvastatin loading efficiency of the polymer PCL-PGS-HA-SIM is (76%).

The drug release mechanism was studied using Korsemeyer-Peppas model which describe drug release from a polymeric system[43].

$$\boldsymbol{M_t/M_n = kt^n}$$

Where $\boldsymbol{M_t/M_n}$the fraction of drug release is at time t, K is rate constant depending on the structural and geometric characteristic of the drug polymer system, n is the release exponent. the n value is used to characterize different release mechanisms. when Korsemeyer-Peppas is applied to thin films the release exponent n=0.5, or n ≤ 0.5correspond to Fickian diffusion release, the value of n in the range of 0.5 < n > 1is related to non-Fickian release or anomalous diffusion this mean that drug release follows both diffusion and erosion mechanisms, and n=1 corresponds to case II where drug release is independent of time.

The Kopcha model is also used to quantify the relative contribution of diffusion and polymer relaxation to drug release[44].

$$\boldsymbol{Q_t = At^{1/2} + Bt}$$

Where A is the diffusional exponent and B is the erosional constant. If A is much greater than B value then the ratio of the exponent A/B will be high suggesting than the drug release from the matrix is primarily controlled by diffusion mechanism and if B value is higher than A value then the ratio of the exponent A/B will be low suggesting than the dominant drug release mechanism is erosion or polymer relaxation mechanism.

2.3 *Invitro* biomimetic mineralization study

The assessments of *in vitro* bio mineralization on the composite nanofibers were carried out in simulated body fluid (SBF) solution. The SBF solution was prepared as reported previously by our group [3]. The samples of different nanofibers with dimension of (2×2 cm) were placed in SBF solution at 37 °C. The SBF solution was replaced every 48 h. SBF-treated samples were rinsed with distilled water and vacuum dried for further analysis for SEM and EDS analysis.

Alizarin res S (ARS) was further used to evaluate the decomposition of calcium compounds on the SBF treated samples, briefly SBF treated samples were washed with distilled water, then fixed in 3.7% buffered formaldehyde for 30 min, and stained with 1 mL of ARS solution (40 mM, pH 4.1) in a 48-well plate for 20 min on an orbital shaker. Samples were then rinsed with distilled water to remove the excess dye, transferred into another well plate, and treated with 50% acetic acid (1 mL) for 30 min. The dissolved dye was diluted with distilled water in a 1:4 ratio, and pH was adjusted to 4.1. A microplate reader (Sunrise Tecan, Austria) used to measure the absorbance of the solution at 562 nm in a 96-well plate.

The SBF solution was replaced every 48 h. SBF-treated samples were rinsed with distilled water and vacuum dried for further analysis. For this work we considered Pure PCL nanofiber mat as control to study effect of adding the nanoparticles alone and composite of the nanoparticles and drug.

2.4 *In vitro* cell culture study

Pre-osteoblast cells (MC3T3-E1), were cultured in α-Minimal Essential Medium (α-MEM, Hyclone) supplemented with 10% fetal bovine serum (FBS, GIBCO) and 1% penicillin-streptomycin in 5% CO_2 at 37 °C with 95% humidified atmospheric condition. Electrospun membranes with 12 mm diameters were placed into 48-well culture plates and sterilized by UV light for 24 h. Then, cell suspensions with a density of 1×10^4 per well were seeded onto sample surfaces and incubated at 37 °C and 5% CO_2 atmosphere until characterization. Cell proliferation was measured based on a Cell Counting Kit-8 (CCK-8, Sigma–Aldrich, Korea) assay. After cultured for 2, 4 and 6 days, each well was refreshed with 50 µL CCK-8 test solution and co-incubated for another 2 h. Then, the final solutions were read for absorbance at 450 nm through a microplate reader (Infinite F50, TECAN, Switzerland).

To examine cell attachment and spreading, the MC3T3-E1 cells were cultured directly on the different samples at seeding density of (10×10^3 cells/well). The one and three-day cell cultured samples were washed with phosphate buffer saline (pH 7.4) and fixed with 2.5% glutaraldehyde for 1 h, followed by washing with 20%, 30%, 50%, 75%, and 95% ethanol for 20 min. They were dried overnight in a laminar flow hood. The cell morphology and attachment were determined via SEM. For this work we considered Pure PCL nanofiber mat as control to study effect of adding the nanoparticles alone and composite of the nanoparticles and drug.

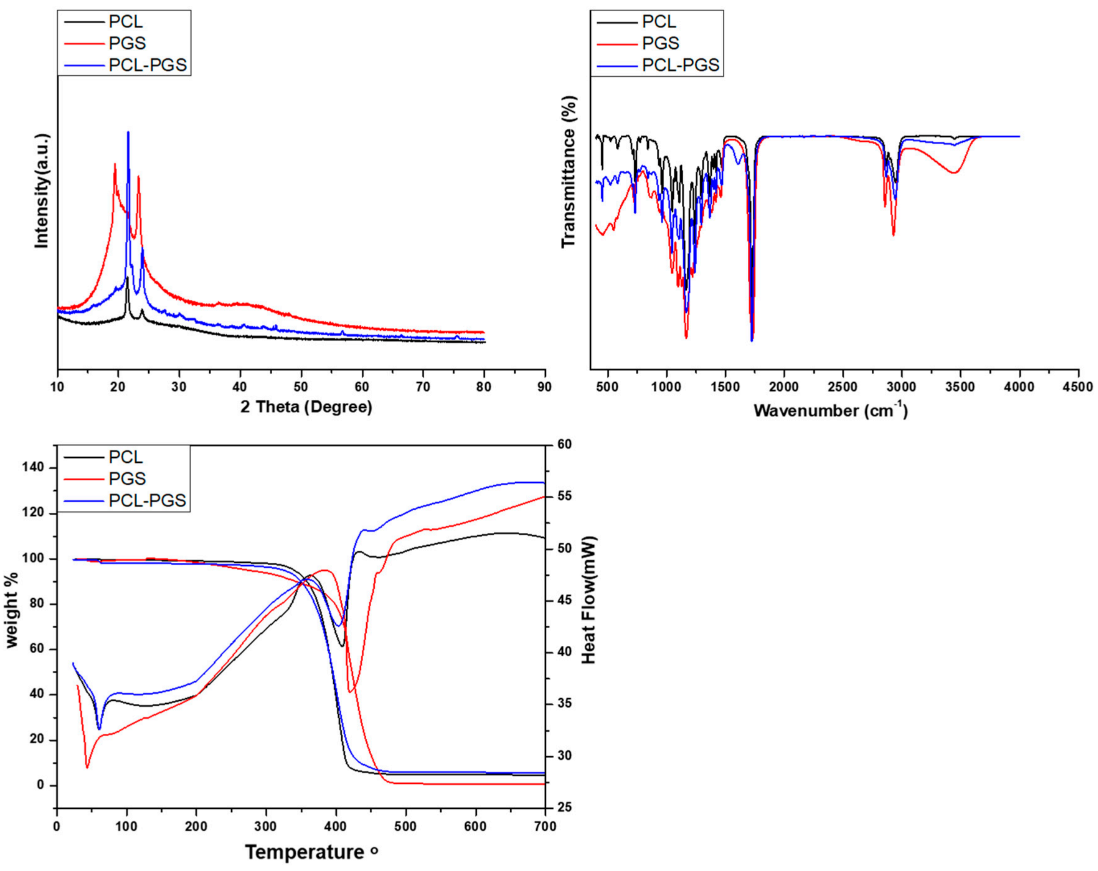

Figure 1. XRD patterns, FTIR spectra and TGA-DSC thermographs of different samples.
